# Chromosomal Heteromorphisms and Cancer Susceptibility Revisited

**DOI:** 10.3390/cells11203239

**Published:** 2022-10-15

**Authors:** Thomas Liehr

**Affiliations:** Institute of Human Genetics, Jena University Hospital, Friedrich Schiller University, 07747 Jena, Germany; thomas.liehr@med.uni-jena.de

**Keywords:** heteromorphism, copy number variation, banding cytogenetics, molecular cytogenetics, cancer, tumor, satellite DNA

## Abstract

Chromosomal heteromorphisms (CHs) are a part of genetic variation in man. The past literature largely posited whether CHs could be correlated with the development of malignancies. While this possibility seemed closed by end of the 1990s, recent data have raised the question again on the potential influences of repetitive DNA elements, the main components of CHs, in cancer susceptibility. Such new evidence for a potential role of CHs in cancer can be found in the following observations: (i) amplification and/or epigenetic alterations of CHs are routinely reported in tumors; (ii) the expression of CH-derived RNA in embryonal and other cells under stress, including cancer cells; (iii) the expression of parts of CH-DNA as long noncoding RNAs; plus (iv) theories that suggest a possible application of the “two-hit model” for euchromatic copy number variants (CNVs). Herein, these points are discussed in detail, which leads to the conclusion that CHs are by far not given sufficient consideration in routine cytogenetic analysis, e.g., leukemias and lymphomas, and need more attention in future research settings including solid tumors. This heightened focus may only be achieved by approaches other than standard sequencing or chromosomal microarrays, as these techniques are at a minimum impaired in their ability to detect, if not blind to, (highly) repetitive DNA sequences.

## 1. Introduction

A high level of genetic variability and diversity among individuals of a given species is typically observed in a genetically healthy vertebrate population. From this point of view, the human species is evolutionarily well-prepared for many challenges to be expected from the environment, and a real-world example is Coronavirus disease (COVID-19) [1,2].

As recently summarized [3], there are several levels of variations present in human genomes. At first, a high variance of single nucleotide differences in coding regions (genes) is eye catching; it leads to different alleles, which may influence the phenotype of an individual [2,4]. Euchromatic regions include dosage-independent genes that may vary in copy numbers without (yet known or identified) influence on the phenotype or health of an individual; these were identified in 2004 and are referred to as copy number variants (CNVs) in the current literature [5,6]. These CNVs are generally only detectable by chromosomal microarray (CMA) studies and the majority of these findings are considered “CNVs detectable by molecular genetics” (MG-CNVs) [7,8]. MG-CNVs can be so sufficiently large that they become visible in banding cytogenetics and termed “cytogenetically visible copy number variations” (CG-CNVs) [7,8]. At present, euchromatic CG-CNVs and MG-CNVs are the focus of intense research [9]. Most of these CNVs are not yet correlated with phenotypic outcomes and/or clinical symptoms, even though many studies have attempted to find correlations for different conditions such as phenotypic variability, complex behavioral traits, disease susceptibility, and predispositions to infections, obesity, and others [10]. For a subset of these CNVs located in specific genomic regions, losses or gains of copy numbers are associated with so-called microdeletion—or microduplication—syndromes (MMS), respectively, where dosage-sensitive genes play a role in disease development [9,11]. However, even the effects of well-established genomic disorders [9]/syndromes are generally hard to predict, given “individual genetic background” considerations [12]. It is more likely a rule than an exception that families are identified as outlined in the following example: there is an index patient with typical symptoms of a given microdeletion syndrome (e.g., DiGeorge syndrome); a typical disease causing deletion del (22) (q11.2q11.2) is identified by a CMA and the diagnosis is established. Subsequently, parental studies show the deletion is maternally inherited, but the mother shows no or only cryptic clinical signs as expected in DiGeorge syndrome. However, the healthy father has a CNV kilo- to mega-base in size within another part of the genome, which is also present in the index patient. Situations such as this suggest that there may be a so-called “two-hit model for euchromatic CNVs”, which could explain different phenotypes within a family [12]. In addition, the unmasking of either a recessive mutation or a functional polymorphism of the remaining allele could be disease-causing for a microdeletion syndrome [13].

**Figure 1:**
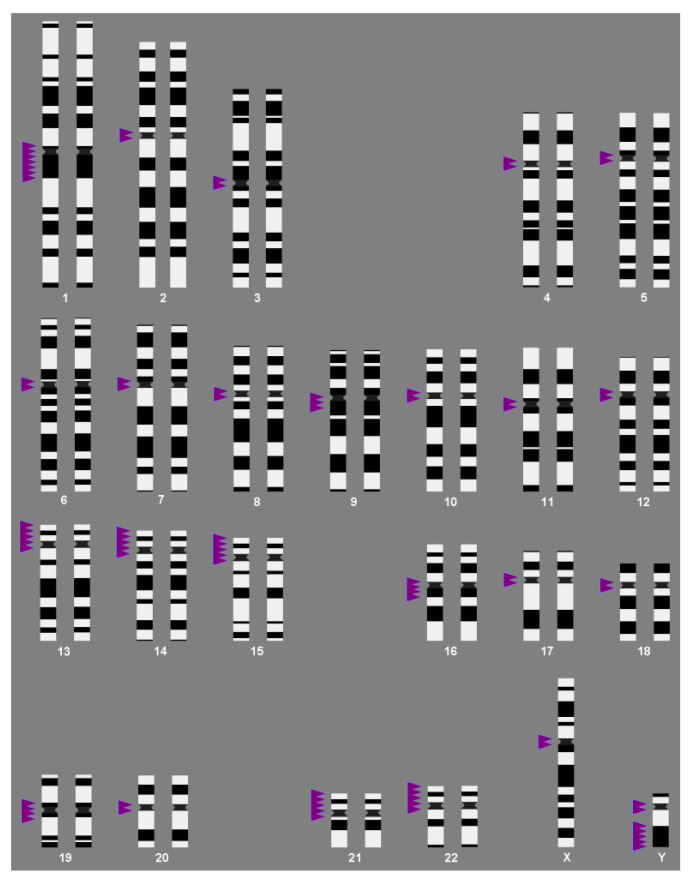
Ideograms of human chromosomes highlighting with arrowheads the regions of heterochromatic, cytogenetically visible copy number variations (CG-CNVs); see also Table 1.

No comparable attention has been given to heterochromatic CNVs when compared to euchromatic CNVs, even though there are many more of them, given they constitute up to 75% of the human genome [8] and have been previously reviewed as disease-causing variations [3]. The tremendous amount of heterochromatic variation in the human genome can be subdivided into many classes: micro- and mini-satellites (also summarized as variable number of tandem repeats (VNTRs), small-scale insertion/inversion/deletion/duplication polymorphisms (SSIIDDs), and small-scale repetitive elements (SSREs—including long interspersed nuclear elements (LINEs), short interspersed nuclear elements (SINEs), polymorphic mitochondrial insertions (NumtS) and higher-order repeats (HOR) of satellite DNAs) [3]. All of these, also called “polymorphic”, are DNA stretches localized along the entire length of all 24 human chromosomes. However, they are normally concentrated in centromeric regions, short arms of acrocentric chromosomes, and in the male Y-chromosome, sub-band Yq12 (Figure 1, Table 1). 

Most of the MG-CNVs variants are below the resolution of light microscopy and thus not assessable by banding cytogenetics [8]. Some of them, however, can be visualized by fluorescence in situ hybridization (FISH) [16]. In addition, there are large heterochromatic CG-CNVs called chromosomal heteromorphisms (CHs), which are not currently considered in any analyses; however, according to cytogenetic data from the 1970s, an average of four to five CHs are present per person [8]. As such, CHs (e.g., of an acrocentric short arm) can achieve an expansion of up to the length of a chromosome 13q; this means instead of ~3096 Mb, an individual would have ~3194 Mb of DNA per cell (calculated acc. to [17]). This is ~3% more DNA than normally present; it is hard to believe this is without any effect on the carrier. 

CHs are defined here as identical to heterochromatic CG-CNVs—best to be visualized in a cytogenetic preparation under a light microscope. However, during recent decades, research interest in banding and molecular cytogenetics (FISH) has decreased in parallel with the rise in molecular genetics [14]. In particular, human genetics is about to forget its two major roots in (i) genetic counselling [18] and (ii) the structure of chromosomes [19,20]. Therefore, it is easy to understand why CHs went out of the research focus. Even though CHs constitute at least 10% of the human genome [21], they became undetectable by application of a CMA and sequencing approaches. Both latter approaches are—due to technical reasons—entirely blind to human repetitive DNA [22] largely identical to CHs, and were lost to study given the saying: “Out of sight, out of mind”. Just recently, a paper demonstrated that the entire genome can be comprehensively sequenced [15], and this may be a landmark for new research on heterochromatic MG-CNVs and heterochromatic CG-CNVs. In Table 1, the available cytogenomic approaches are compared with respect to their abilities to access (potentially) heterochromatic DNA stretches (see also [14]). 

## 2. Large Heterochromatic CG-CNVs/Chromosomal Heteromorphisms

This review paper focuses on CHs, large heterochromatic CG-CNVs, as summarized in a corresponding database [23]. Possible variants may affect regions listed in Table 2. Please note, sizes attributed to the heterochromatic regions in genomic builds are not found in the literature data, as detailed elsewhere [8]. As an example: there is no evidence from FISH or literature data [24] that the centromeric region of the Y-chromosome should be only 0.3 Mb in size, while all other centromeric regions are suggested to be in the range of ~3 to ~5 Mb, as presently stated in the UCSC browser [17]. Additionally, many long-established satellite sequences, such as D12Z3 and D17Z1 for alpha satellite sequences at centromeres 12 and 17, remain absent from genomic browsers still [8,17]. 

**Figure 2:**
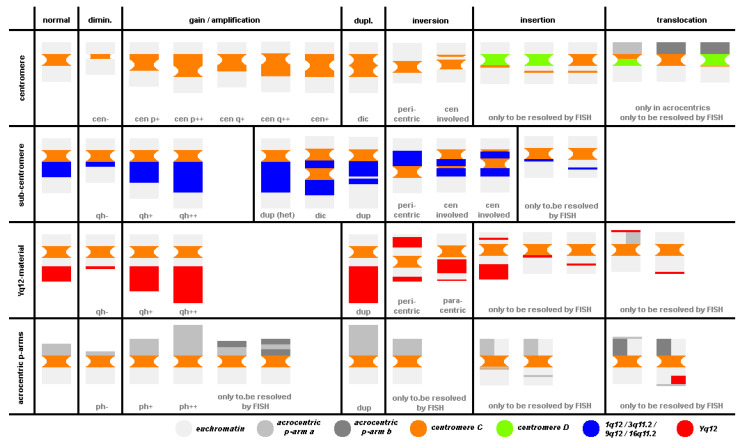
Heterochromatic CG-CNVs can lead to different deviations in chromosome shape and size. Centromeres (line 2), subcentromeric regions—mainly 1q12, 3q11.2, 9q12, and 16q11.2 (line 3), band Yq12 (line 4), and the short arms (p-arms) of the acrocentric chromosomes (line 4) can be involved. The normal status is presented in column 2; reduction in size (dimin.), gain/amplification, duplications (dupl.), inversions, insertions, and translocations of heterochromatic material are shown in the following columns. The colors represent different chromosomal regions as explained in the legend at the bottom of the figure.

The basic types of variations, which can be observed in heteromorphic regions of the human genome (CHs), are summarized in Figure 2 (for more details, see [23]; nomenclature acc. to ISCN 2020 [25]).

- There can be size variants of the centromeres of all 24 human chromosomes as diminished (=cen−) or enlarged by amplification or unequal crossing over (=cen+, cen++), or due to a duplication leading to a (pseudo) dicentric derivative. As enlarged centromeric regions can also include inversions, such events are also included here as common CHs. Centromeric insertions in other centromeres and/or euchromatic material can lead to altered banding patterns in cytogenetic analyses within healthy individuals. However, such derivative chromosomes can only be elucidated and characterized by FISH. Finally, centromeres of acrocentric chromosomes may provide unexpected FISH results when using alpha-satellite-specific probes; here, rare, unbalanced translocation events exclusively involving heterochromatic material may be the reason for CHs, again only resolvable by FISH.
- Similar to centromeres, subcentromeric heterochromatic blocks of chromosomes 1, 3, 9, and 16 (1q12, 3q11.2, 9q12, and 16q11.2) may by diminished or enlarged in size. However, here, amplification of material cannot be distinguished from duplication. Moreover, in chromosome 9, so-called hemi-heterochromatic bands adjacent to 9p11.1 (9p11.2~12) and to 9q12 (9q13) comprise copy-number-independent regions involved in euchromatic variants [26]. Thus, here, many subtypes of CHs consisting of hetero- and euchromatin are regularly observed in banding cytogenetics. Heteromorphic inversions and insertions of this region are also present in the human population.
- In males, the sub-band Yq12 and satellite DNAs are normally gender-specific. Sub-band Yq12 can also be reduced to minimal size or amplified to dramatically large sizes without obvious phenotypic effects. Amplifications and duplications, as well as inversions and insertions, can only be reliably characterized by FISH. However, Yq12 material can be transferred to other autosomes or even an X chromosome, and then also be observed throughout generations in males and females [27].
- There are 10 acrocentric chromosomes in the human genome, which carry nearly identical short (p-) arms. They comprise only one identified, substantially important genetic material, the nucleolus organizing region (NOR), each. As in many other vertebrate species, one NOR-bearing chromosome pair is sufficient for a species to function [28], and the tremendous variability observed for these 10 regions is no surprise. Figure 2 includes many of these variants, which can be picked up or at least suggested based on banding cytogenetics. FISH enables detection and substantially more insights as summarized elsewhere [8,23].

Overall, 250 heterochromatic CHs have been reported [23] and are an expression of the variability in heterochromatic DNA in the human genome, which is visible in light microscopy. CHs can be easily accessed and further analyzed by molecular cytogenetics; also, novel sequencing approaches and algorithms could be applied to resolve them in more detail, but are rarely used [22,29]. New insights on the evolution and variance are on our doorstep ready to be discovered; still, there is no interest at present to invest money, time, and greatness of mind to conquer this undiscovered land. Maybe some thoughts on these understudied parts of the human genome and their potential connections to tumorigenesis can stimulate some research towards this area. This seems to be quite timely, as findings in 2018 designated heterochromatin the “guardian of the genome” [30].

## 3. Chromosomal Heteromorphisms (CHs) and Cancer

### 3.1. Correlations Based on Pure Banding Cytogenetics

Considering currently available approaches and insights, it seems to be relatively clear that simple, poor banding cytogenetic analyses are not sufficient to yield meaningful clues about variation in CH size and associated clinical consequences. A good example is the so-called Christchurch (Ch1) chromosome, which was suggested to be found as a disease-specific, acquired aberration in chronic lymphocytic leukemia. However, it turned out that the Ch1 chromosome was simply a normal variant of chromosome 21, in which the short arm was (almost) lost [31]. 

Besides this, heteromorphisms of chromosome 9 are another example of the many misleading correlations previously made based on pure banding cytogenetic data. As summarized elsewhere [32], CHs of chromosome 9 were aligned with cancer predisposition and infertility, mental retardation, schizophrenia, the Walker–Warburg syndrome, and the oculo-auriculo-vertebral (Goldenhar) spectrum. None of these correlations could be verified (see, e.g., for infertility [26]). Overall, even though some studies showed surprisingly high concordance rates of a malignancy and special CG-CNV and/or heteromorphic inversions [33], there were always other studies that could not substantiate those specific findings [34].

### 3.2. Possible Correlations

Nonetheless, banding, as well as molecular cytogenetics data, has made heterochromatic CG-CNVs, at the least, suspicious for a role in cancer progression, or it may even be a critical element of initiation. 

#### 3.2.1. Amplification of CHs in Tumors

**Figure 3:**
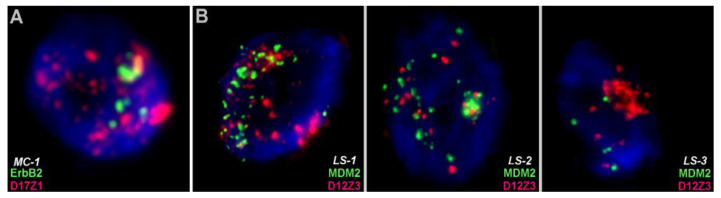
Two color fluorescence in situ hybridization (FISH) results on formalin-fixed paraffin-embedded nuclei derived from (**A**) mammary carcinoma (MC-1) or (**B**) liposarcoma (LS) cases 1 to 3 are presented. In (**A**) ~6 ErbB2 signals (green) with >30 signals of D17Z1 (red—amplification). In (**B**) cases, LS-1 and LS-2 appear to demonstrate a homogeneously staining region, each including between ~10 and ~15 MDM2 (green) and D12Z3 copies (red) each. Case L-3 has an amplification of D12Z3 signals and only 4 MDM2 signals.

Amplification of centromeric, specifically alpha-satellite, DNA is repeatedly observed in tumor cell lines [35,36,37,38] but also primary tumors [39,40], a fact that is most certainly underreported. In our unpublished study, mammary carcinoma samples showed amplification of D17Z1 sequences in 1/414 (=0.24%) and an amplification of D12Z3 sequences in 3/437 (=0.69%) liposarcoma cases. It must be considered here that these amplifications are only picked up by chance; the centromeric probe is simply used as control for chromosome enumeration in parallel with an oncogene-targeting probe. Herein, for example, the target sequences of *ErbB2* in 17q12 and *MDM2* in 12q15 were routinely used in FISH tests to evaluate copy number variation. The centromeres are ~7 and >30 Mb away from the target probes, respectively. A co-amplification with the oncogene in case of MDM2 is rather unlikely, and in MC-1 and LS-3 cases, only alpha-satellite amplification was observed (Figure 3); interestingly, similar observations were previously reported [38,41]. FISH tests targeting only one of the 24 human centromeres identified amplifications of these regions in ~0.5% of the cases in the examples shown herein (Figure 3). By simple extrapolation, it is not a stretch too far to simply multiply 0.5% by 24, which suggests that alpha satellite amplification could be found in ~10% of solid tumors. 

In addition, there are also coincidental reports on cancer-associated amplification and/or imbalanced rearrangements involving other repetitive DNAs, such as Yq12 [42,43], 1q12 [44,45,46,47], and 9q12 [48,49]; in addition, mutations [29] and acrocentric p-arms have also been reported [29,35,50,51,52,53,54,55,56]. 

To understand why these amplicons have not caught more attention, one needs to consider two points: (i) Solid tumor cells are hard to cultivate in culture; as living and dividing cells are a prerequisite to prepare chromosomes, tumor cytogenetic data in solid tumors are scarce [57]. (ii) To obtain (molecular) cytogenetic information from tumors, comparative genomic hybridization (CGH) was established in 1992 [58] and array CGH (aCGH) was deduced from CGH in the early 2000s [14]. However, CGH/aCGH is, as with NGS, not able to provide information on repetitive DNA. Accordingly, amplification of CG-CNVs has not been studied in more detail yet. Still, some papers already propose that cancer-associated alteration of pericentromeric heterochromatin may contribute to chromosome instability [59]—not only in humans, but also in cat [60], rat [61], or mouse genomes [62,63].

#### 3.2.2. Epigenetic Changes in CHs in Tumors

Epigenetic changes (and mutations) in chromatin proteins have also been correlated with cancer progression [30,64]. In addition, epigenetic regulation of centromere chromatin stability by environmental factors has been reported [65]. This connection is of interest given cancer-associated abnormal methylation patterns have been seen in CHs such as hypomethylation in 1q12 [66,67,68,69,70] or 9q12 [71]. In addition, satellite II and III (HSATII and HSATIII) sequences (~5 to 26 bp repeats) have been linked to the heat-shock response and nuclear stress bodies. HSATIII is mainly located in 9q12, while HSATII can be found in 1q12; 6q11, the centromeres of chromosomes 2, 5, 7, 10, 13, 14, 15, 16, 17, 21, 22, and Y, plus all acrocentric short arms [72]. Still, it must be noted that such data are now only available for short satellite DNA repeats; for longer, i.e., alpha-satellite repeats, organized in HOR units, data are still scarce and, in parts, contradictory [73]. 

#### 3.2.3. CHs Expressed on RNA Level

Nearly two decades ago, enhanced expression rates of heterochromatic DNA as RNA (however, only restricted to short HSATII and HSATIII sequences) located in CHs were discovered for cells under stress and in cancer cells [66,74,75,76]. It is now clear that HSATIII can be (over)expressed as so-called long noncoding RNAs (lncRNA) in cancer cells [77,78]. Even the primary influence of lncRNA derived from HSATIII on cancer outcomes have been recently described [79]. Furthermore, in mice, heterochromatic repeats (including alpha-satellites) are transcribed for normal cell function [80].

#### 3.2.4. Is the Two-Hit Model Also Applicable for Heterochromatic CNVs/CHs?

The two-hit model for euchromatic CNVs, as suggested in 2010 [12], has already been supported as potentially valid, and maybe at a lower efficiency, is valid also for heterochromatic CNVs—especially for heterochromatic CG-CNVs [7]. The majority of constitutional syndromes in humans are suggested to result from multigenic traits [81]. Interestingly, a multigenic disorder is defined to be, in part, genetic predisposition, and at the same time, it is emerging that environmental conditions support transformation to disease—this includes cancer development. Furthermore, there are many inherited diseases, as previously discussed for MMS, where the ‘genetic background’ of the patient alters the expression of a disease. Thus, according to the present state of research, an influence of the factor ‘size and composition of CHs’, either via the “two-hit model” and/or via other mechanisms such as expression levels of lncRNAs, cannot be excluded as major factors in disease. 

#### 3.2.5. Necessary Future Research Directions

Yet, only a small number of cancer-related studies have focused on lncRNAs derived from small repetitive DNAs, and none have taken into account lncRNAs or the size and constitution of heterochromatic/satellite DNA located in CHs. This provokes a number of questions.

- Should other repeats/longer satellite sequences/HORs be studied, especially in cases of cancer?
- Additionally, might consideration being given to the individual sizes of heterochromatin make sense? If DNA stretches located in CHs matter for normal cell function, as shown in our favorite animal model—the mouse [80]—would it not to be expected that differences in the proportion of CHs compared to overall stable euchromatic genome size would result in some effects?
- What about studies checking on the cancerogenic effects of two types of cell lines: those with a high proportion of heterochromatin due to large CH regions and those with smaller, almost absent CHs?
- Why not combine banding cytogenetic data available for CHs in leukemia and lymphoma with data on lncRNA derived from HSATIII in the same cells as the basis for further studies?

## 4. Conclusions

Overall, it must be stated that the question of which role CHs may play in cancer remains unanswered. There are hints that lncRNAs derived from these regions have an influence on tumorigenesis. Still, there is another consideration for future research discoveries: we must remember the advantages, benefits, and restrictions of each of the currently available cytogenomic techniques (see Table 1). Only studies accessing, in parallel, the chromosomal level (through banding and/or molecular cytogenetics), the DNA level (NGS, using new algorithms to also access highly repetitive sequences), the RNA level (NGS, maybe also using the aforementioned algorithms so the technology is not blind for subgroups of lncRNAs), and other techniques occasionally used (such as immunohistochemistry) [78] need to be combined. No single approach can be disregarded or declared outdated if this question shall be fully assessed.

## Figures and Tables

**Table 1:** Overview on cytogenomic approaches and their ability to access heterochromatic CNVs based on technology (Table according to information from [14]).

	Can Access Heterochromatic CNV				
Cytogenomic Approach	SNPs	MicS/MinS	SSREs	SSIIDDs	CHs
banding cytogenetics	-	-	-	(+)	+
molecular cytogenetics	-	+	+	+	+
(Southern) blotting	+	+	(+)	+	(+)
Pulsed-field gel electrophoresis	-	+	(+)	+	(+)
microsatellite analyses	(+)	+	-	-	-
CMA	-	-	(+)	(+)	-
Sanger sequencing	+	(+)	+	(+) ^1^	-
NGS	+	(+)	+	(+) ^2^	-
optical genomic mapping	-	-	-	(+)	(+)

^1^ sequencing of cloned repetitive elements [8]; ^2^ workup of sequencing data with special algorithms [15]. Abbreviations: - = no; + = yes; (+) = yes—under special conditions detectable; CHs = chromosomal heteromorphism; CMA = chromosomal microarray; MicS = micro-satellites; MinS = mini-satellites; NGS = next-generation sequencing; SNPs = single-nucleotide polymorphisms; SSIIDDs = small-scale insertion/inversion/deletion/duplication polymorphisms; SSREs = small-scale repetitive elements.

**Table 2:** Regions being affected by/involved in heterochromatic CG-CNVs.

Cytoband	Position [GRCh38/hg38]
1p11.1–q11	121,700,001–125,100,000
1q12	125,100,001–143,200,000
2p11.1–q11.1	91,800,001–96,000,000
3p11.1–q11.1	87,800,001–94,000,000
3q11.2	94,000,001–98,600,000
4p11–q11	48,200,001–51,800,000
5p11–q11.1	46,100,001–51,400,000
6p11.1–q11.1	58,500,001–62,600,000
7p11.1–q11.1	58,100,001–62,100,000
8p11.1–q11.1	43,200,001–47,200,000
9p11.1–q11	42,200,001–45,500,000
9q12	45,500,001–61,500,000
10p11.1–q11.1	38,000,001–41,600,000
11p11.1–q11.1	51,000,001–55,800,000
12p11.1–q11.1	33,200,001–37,800,000
13p13–p11.2	1–16,500,000
13p11.1–q11	16,500,001–18,900,000
14p13–p11.2	1–16,100,000
14p11.1–q11.1	16,100,001–18,200,000
15p13–p11.2	1–17,500,000
15p11.1–q11.1	17,500,001–20,500,000
16p11.1–q11.1	35,300,001–38,400,000
17p11.1–q11.1	22,700,001–27,400,000
18p11.1–q11.1	15,400,001–21,500,000
19p11–q11	24,200,001–28,100,000
20p11.1–q11.1	25,700,001–30,400,000
21p13–p11.2	1–10,900,000
21p11.1–q11	10,900,001–13,00,000
22p13–p11.2	1–13,700,000
22p11.1–q11.1	13,700,001–17,400,000
Xp11.1–q11.1	58,100,001–63,800,000
Yp11.1–q11.1	10,300,001–10,600,000
Yq12	26,600,001–57,227,415

## Data Availability

All data supporting the reported results can be found in this paper.
